# The Role of Ultrasound as a Diagnostic Tool for Sarcopenia

**DOI:** 10.14283/jfa.2018.24

**Published:** 2018-08-13

**Authors:** Howard J. Stringer, D. Wilson

**Affiliations:** 1 College of Medical and Dental Sciences, University of Birmingham, B15 2TT, Edgbaston, UK; 2 Heart of England NHS Foundation Trust, Birmingham, UK

**Keywords:** Sarcopenia, ultrasound, diagnosis

## Abstract

Sarcopenia is the progressive loss of skeletal mass and strength, particularly in older adults, with consequent reduction in function and independence. Changing population demographics, have resulted in increased prevalence of sarcopenia and this is associated with a considerable economic burden. Whilst simple, effective, non-intrusive management of this condition exists, no routine diagnosis takes place either in the UK or in many other countries, partly due to an absence of pragmatic clinical diagnostic tools to support the early identification of the syndrome. This position paper aims to provide a short overview proposing the potential case for developing ultrasound as a new and alternative diagnostic tool for identifying sarcopenia.

## Introduction and Background

Sarcopenia is defined as ‘a syndrome of progressive and generalised loss of skeletal muscle mass and strength with a risk of adverse outcomes such as physical disability, poor quality of life and death' by the European Working Group on Sarcopenia in Older People (EWGSOP) (1). The three core criteria of low muscle mass, low muscle strength and poor physical performance can be used both in the diagnosis of sarcopenia and in the assessment of its severity, with low muscle mass alone indicating pre-sarcopenia; associated loss of muscle strength or performance indicating sarcopenia; and the presence of all three conditions indicating severe sarcopenia. The International Working Group on Sarcopenia (IWGS) have described similar diagnostic criteria without the hierarchical approach defining it as poor physical performance and the presence of low muscle mass (2). In contrast to the definition used by EWGSOP, the IWGS considers muscle strength as a preliminary indicator of sarcopenia rather than definitive to diagnosis.

Sarcopenia is primarily, though not exclusively, a disease of older adults (1) and can lead to multiple poor outcomes including frailty, disability, loss of independence and reduced quality of life (3). Prevalence estimates in the UK suggest that around 4.6% of males and 7.9% of females are affected (4), while in the USA, comparable population studies indicate that around 36.1% of older adults are affected (5). The variances between these two estimates are considerable and highlight the inconsistencies in diagnostic methods and algorithms used. A systematic review of 35 articles (totalling 58404 communitydwelling participants aged 60 years and older) identified an overall prevalence of 10% in both men and women; this review also identified that the prevalence of sarcopenia was almost double in non-Asian individuals compared to Asians when Bioelectrical Impedance Analysis (BIA) was used (6). Within UK clinical settings, around 14-33% of those in long-term care were assessed to have sarcopenia (3). Global estimates suggest that around 50 million individuals currently have sarcopenia, this is estimated to rise to 200 million over the next 40 years (1). The clinical, personal and economic implications of sarcopenia are therefore substantial. The direct cost burden to the health and social care budget in the United States in 2000 was estimated to be approximately $18.5 billion per annum (7), represented by hospitalisation, nursing home admissions and home healthcare expenditure; this is probably an underestimate of the true healthcare cost.

Despite the adverse impact of sarcopenia on the individual and their carers, the condition remains poorly understood and inconsistently diagnosed and managed. Diagnostic identification of low muscle mass tends to be complex, time-consuming and costly (1, 2, 8). Once sarcopenia is diagnosed, the therapeutic approach is typically conservative, management is primarily dietary supplements and physical activity (9). The limited evidence available demonstrates these interventions can improve strength and function, improve quality of life and consequently reduce the economic burden (9, 10, 11, 12, 13, 14). Whilst management is currently typically conservative there are ongoing clinical trials investigating the benefits of pharmacological approaches such as the effect of ACE inhibitors (14).

The foregoing resume suggests that the current effective management of sarcopenia is currently non-invasive and relatively low-cost. This makes the case for a more proactive approach to intervention, since if sarcopenia is diagnosed, it can be cheaply and successfully managed, but if it remains undiagnosed, its impact can be progressively debilitating. The argument for routine diagnosis and management would seem, therefore, to be unassailable. However, a key impediment to the introduction of wide scale diagnosis relates to the current diagnostic procedures. The diagnostic algorithm suggested by the EWGSOP assesses muscle mass, strength and performance. However, many of the relevant measurement techniques for measuring muscle mass have numerous flaws that make them unsuitable for routine use in frail older adults. Alongside the standard reliability and validity problems, there may also be poor accessibility for frail older adults, be only suitable for certain settings and can therefore be incompatible with the target population. For example, muscle mass measurement and calculation methods, while abundant, can also consider different outcomes. Depending on the device used, BIA can calculate total lean mass (body weight minus body fat), appendicular lean muscle mass (aLM - lean mass in the limbs) or both (15). To further complicate matters, these two measurements can be estimated using computed tomography (CT), magnetic resonance imaging (MRI), BIA and dual-energy x-ray absorptiometry (DXA) (1, 2, 3, 16). The benefits and limitations of the available measurement techniques are summarised in Table 1.

**Table 1:** Benefits and Limitations of different modalities used in estimation of skeletal muscle mass

Modality	Benefits	Limitations
MRI	• No ionising radiation	• Expensive
	• Good for imaging soft tissues	• Time consuming
	• Able to review images after scanning	• Limited accessibility for frail community based patients and those with cognitive impairment
	• Thorough image acquisition	• Confined space in scanner
		• Limited availability
		• Cannot use if patient has metal work/some pacemakers
		• Requires interpretation by radiologist
CT	• Able to review images after scanning	• Expensive
	• Thorough image acquisition	• Radiation exposure
		• Time consuming
		• Poor accessibility
		• Confined space in scanner
		• Limited availability
		• Requires interpretation by radiologist
DXA	• Can also identify bone mineral density	• Expensive
	• Radiation exposure is small	• Radiation exposure
		• Time consuming
		• Poor accessibility
BIA	• Safe	
	No radiation exposure	• Dependent on hydration status
	• Quick to perform	• No assessment of reliability in dependent oedema, congestive cardiac failure and renal failure
		• No reliability data in frail older adults
		• Not universally portable
		• Cannot use if patient has metal work or electronic device implants
		• Varying accuracy between machines
Ultrasound	• Extremely safe	• Variety of probes required to achieve varying depth/resolution
	• No ionising radiation	• Limited use in obese patients
	• Ability to perform dynamic testing	• Quality and interpretation of images is user dependent
	• Portable	• No criteria for diagnosis of low muscle mass
	• Cost-effective	
	• Low-risk	
	• Quick to perform	
	• Suitable in all patient groups	
	• Can be interpreted at bedside by a lay sonographer	

The synopsis presented in Table 1 suggests that, on balance, ultrasound may offer the most promising option for routine diagnosis particularly when considering a community based frail older adult population. There are both physical and cognitive limitations to the accessibility of MRI, CT and DXA in a frail older adult population which are not applicable to bedside tests such as ultrasound or BIA. However, BIA is dependent of hydration status and unlikely to be accurate in states of peripheral oedema.

## Towards a new diagnostic procedure

When the target sarcopenic population is typically frail, elderly and immobile, the imaging technique must be easily accessible, both geographically and physically, and in this regard ultrasound is clearly superior to the other methods outlined in Table 1. In particular, it offers a non-invasive, portable and safe imaging modality, whilst having the additional benefits of: maintaining image clarity, being widely used in medicine, familiar to clinicians, reliable and easily interpreted by the lay sonographer (17, 18). More specifically, the evidence-base for the use of ultrasound in the measurement of the thickness of numerous muscle groups is convincing (17, 19, 20, 21, 22, 23), supporting accurate and reliable depth measurements across different muscles and populations. However, ultrasound does pose some problems, which although not insurmountable, would need further development before its use as a routine diagnostic tool could be considered. In particular, there is currently a lack of a clear and standardised protocol for the assessment of skeletal muscle, including: no widely established norms for the various muscle thicknesses within the nonclinical population; there is no definitive agreement either about which muscle group should be measured or the probe site; or defined criteria for low muscle mass identification in sarcopenia. The heterogeneity of methods that could be adopted for muscle mass measurement and analysis could impact on future clinical and research implementation, thus it is imperative that a standardised and easily applied technique is agreed on.

Notwithstanding these concerns, there is evidence that ultrasound is an excellent surrogate marker of aLM (1, 23, 24, 25, 26, 27). Using regression analysis, measures of muscle depths can be used to predict overall skeletal muscle mass (23, 24, 28, 29, 30); aLM data reliably correlate with those derived from other measurement techniques, such as DXA-derived aLM scores in older adults (26). Taken together, these findings suggest that ultrasound is as accurate and reliable as DXA-derived aLM data, but is more easily obtained (26). While a composite measure of skeletal muscle mass is likely to be more accurate (26), this is not always feasible in a frail elderly population, and therefore a simpler approach such as ultrasound measurement of muscle thickness may be superior in this population (29). Abe et al have suggested that ultrasound measures of forearm muscle thickness may be a useful measure in sarcopenia (26). They have shown that forearm thickness is strongly correlated with hand grip strength, suggesting a positive association with physical activity levels; this clearly requires further testing, but it has clinical appeal.

**Figure 1:**
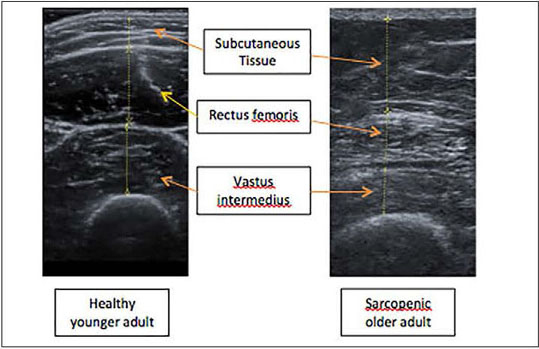
Ultrasound echogenicity of healthy younger and sarcopenic older adult

Furthermore, ultrasound can measure changes in muscle architecture and composition such as muscle echogenicity, pennation angle and fibre length (17). Muscle strength and physical performance, the other defining criteria of sarcopenia, are affected by not only muscle size but muscle quality (1). Muscle quality is defined as muscle function (strength or power) per unit of muscle size (mass or cross-sectional area) (20, 22, 31) and is affected by: the morphological characteristics of the muscle, aerobic capacity, intramuscular adipose tissue, fibrous tissue and motor units. It has been suggested that ultrasound can be used to determine muscle quality (32, 33). Ultrasound provides scope for the efficient assessment of muscle quality whilst providing insight into the pathophysiology of sarcopenia in addition to the diagnosis of low muscle mass. Previous research has demonstrated that echogenicity increases with age, whilst the pennation angle of muscles decreases (17, 34, 35). The relationship between muscle size, quality and function is as yet incompletely characterised and a move to assessing both size and quality simultaneously would improve our understanding. p ]Ultrasound echogenicity has been particularly highlighted as a technique to measure muscle quality. Ultrasound echogenicity refers to the capacity of any tissue to reflect and absorb ultrasound waves, and can be calculated using grayscale analysis derived from ultrasound imaging (36). In essence, the whiter the image the higher the proportion of slow-twitch muscle fibre and intra-muscular adipose tissue; high ultrasound echogenicity values have been correlated with lower muscle quality and grip strength (21, 22, 37). An example of the differences in ultrasound echogenicity in sarcopenic older adults compared to healthy younger adults is depicted in Figure 1. This aspect of the ultrasound image can therefore be used as an index of muscle quality. The correlation of intramuscular adipose tissue, as demonstrated by grayscale analysis, with various muscle performance measures, such as grip strength and walk speed, needs to be more thoroughly assessed; if their association can be reliably established, then the value of ultrasound imaging in sarcopenia diagnosis is further strengthened. Whilst a direct relationship between adipose infiltration and muscle weakness is not yet fully understood, there are several potential mechanisms explaining the phenomenon (38, 39, 40, 41). As there are many factors contributing to muscle strength, echogenicity analysis of muscles may offer an insight into the pathophysiology of sarcopenia rather than act as a direct measurement technique.

## Recommendations and conclusions

The foregoing overview suggests that ultrasound may be a valuable potential diagnostic tool enabling the easy routine diagnosis of low muscle mass. While there are several obstacles (summarised above) that need to be negotiated before ultrasound can be confidently adopted into clinical practice, it is undoubtedly the case that the current gold standards of muscle mass measurement may be neither accessible nor suitable for assessing the frail older adult. What is clearly required is a pragmatic diagnostic tool that is: simple, easily and unambiguously interpreted, non-invasive, poses no risk, and can be used within both community and hospital settings. Ultrasound meets these requirements and offers portability, cost-effectiveness and speed in addition. However, while its use appears promising, the evidence-base needs to be established and a standardised protocol developed. In-depth comparisons with other gold-standard data are essential to establish its validity and reliability, while a normative data-set for the creation of low muscle mass criteria also needs to be collated. Forearm muscle depth could be an especially valuable tool as a diagnostic technique that does not require removal of clothes or transferring of individuals.

Ultrasound might be an important addition to the diagnostic tool-box, allowing quick and early diagnosis of sarcopenia and facilitating appropriate dietary and exercise interventions. Besides the obvious benefits to the individual and the carer, the cost implications for the health service are minimal, while the savings are considerable. The potential value of ultrasound muscle screening for older adults and their care providers is worthy of further consideration and investigation.

*Funding:* Dr. Wilson reports grants from Medical Research Council - Arthritis Research United Kingdom, during the conduct of the study. The sponsor had no role in the preparation of the manuscript or in the review and approval of the manuscript.

*Conflicts of Interest:* No conflicts of interest from the authors.
